# Commensal *Escherichia coli* Strains Can Promote Intestinal Inflammation via Differential Interleukin-6 Production

**DOI:** 10.3389/fimmu.2018.02318

**Published:** 2018-10-09

**Authors:** Hatem Kittana, João Carlos Gomes-Neto, Kari Heck, Abby L. Geis, Rafael R. Segura Muñoz, Liz A. Cody, Robert J. Schmaltz, Laure B. Bindels, Rohita Sinha, Jesse M. Hostetter, Andrew K. Benson, Amanda E. Ramer-Tait

**Affiliations:** ^1^Department of Food Science and Technology, University of Nebraska-Lincoln, Lincoln, NE, United States; ^2^Department of Veterinary Pathology, Iowa State University, Ames, IA, United States

**Keywords:** commensal *E. coli*, intestinal inflammation, interleukin-6, strain-specific immune responses, gnotobiotic mice

## Abstract

*Escherichia coli* is a facultative anaerobic symbiont found widely among mammalian gastrointestinal tracts. Several human studies have reported increased commensal *E. coli* abundance in the intestine during inflammation; however, host immunological responses toward commensal *E. coli* during inflammation are not well-defined. Here, we show that colonization of gnotobiotic mice with different genotypes of commensal *E. coli* isolated from healthy conventional microbiota mice and representing distinct populations of *E. coli* elicited strain-specific disease phenotypes and immunopathological changes following treatment with the inflammatory stimulus, dextran sulfate sodium (DSS). Production of the inflammatory cytokines GM-CSF, IL-6, and IFN-γ was a hallmark of the severe inflammation induced by *E. coli* strains of Sequence Type 129 (ST129) and ST375 following DSS administration. In contrast, colonization with *E. coli* strains ST150 and ST468 caused mild intestinal inflammation and triggered only low levels of pro-inflammatory cytokines, a response indistinguishable from that of *E. coli*-free control mice treated with DSS. The disease development observed with ST129 and ST375 colonization was not directly associated with their abundance in the GI tract as their levels did not change throughout DSS treatment, and no major differences in bacterial burden in the gut were observed among the strains tested. Data mining and *in vivo* neutralization identified IL-6 as a key cytokine responsible for the observed differential disease severity. Collectively, our results show that the capacity to exacerbate acute intestinal inflammation is a strain-specific trait that can potentially be overcome by blocking the pro-inflammatory immune responses that mediate intestinal tissue damage.

## Introduction

The mammalian gut harbors a wide variety of bacteria (i.e., the gastrointestinal microbiota) that coexist in a mutually beneficial state with their host (1). In this symbiotic relationship, the microbiota contributes to nutrient degradation and metabolism, acts as a barrier that confers resistance to pathogen colonization and helps develop and maintain proper immune responses (2–4). In return, the host provides the niches and nutrients essential for microbial propagation and population survival. Disruption of this homeostatic relationship, which occurs during enteric infection or inflammation, can result in robust and potentially pathogenic immune responses that are thought to drive disease pathogenesis (5). This altered state of homeostasis often manifests as a contraction of the highly-abundant obligate anaerobic bacteria belonging to the Firmicutes and Bacteroidetes and a corresponding outgrowth of Proteobacteria, including members of family *Enterobacteriaceae* (6–8).

Among all *Enterobacteriaceae, Escherichia coli* is the most widely-found facultative anaerobe in mammals (9). A considerable degree of phylogenetic diversity has evolved within the species *E. coli*, with some lineages acquiring various combinations of virulence genes that enable them to adopt overtly pathogenic lifestyles (10). Consequently, populations of *E. coli* can engage in a variety of host interactions, ranging from virulence to mutualism and even protection against infection (11, 12). Even among lineages not known to be primarily pathogenic, phenotypes can also range from the capacity to protect against invading enteric pathogens to exacerbation of inflammation induced by a variety of stimuli (11). With respect to the former, the gut isolate *E. coli* EM0 strain and even the JM105 cloning host strain can each protect against *Salmonella* Typhimurium infection in germ-free mice (13). *E. coli* Nissle 1917 was also reported to protect against *S*. Typhimurium infection, specifically by competing for iron availability (14). In contrast, the pathobiont *E. coli* LF82 can function as a benign member of the microbiota, but also has the capacity to exacerbate intestinal inflammation in TLR5^−/−^ mice (15). These contrasting traits of protection versus pathobiont have not been systematically studied across the population structure of *E. coli*.

Indeed, what differentiates a strain with pathobiont characteristics (such as LF82) from those without is not well-understood. Moreover, only a very small number of isolates have been characterized with pathobiont phenotypes. To gain insight into the strain-specific nature of the pathobiont phenotype, we studied four gut commensal *E. coli* strains isolated randomly from healthy conventional microbiota mice. All isolates were derived from independent animals and, by genotyping, represented independent multi-locus sequence typing (MLST) lineages. When these strains were introduced into gnotobiotic C3H/HeN mice bearing the altered Schaedler flora (ASF), subsequent treatment with the inflammatory stimulus dextran sulfate sodium (DSS) revealed that only two of the strains tested had the capacity to exacerbate inflammatory responses. Specifically, CEC-1 (ST129) or SWW33 (ST375)-colonized mice developed severe intestinal disease characterized by increased production of the pro-inflammatory cytokines granulocyte-macrophage colony-stimulating factor (GM-CSF), interleukin-6 (IL-6), and interferon-gamma (IFN-γ). In contrast, mice colonized with CEC-7 (ST150) or CEC-8 (ST468) experienced only mild or no intestinal inflammation that was comparable to DSS-treated control mice harboring no *E. coli*. Importantly, neutralization of IL-6 production *in vivo* ameliorated *E. coli* ST375-mediated disease. Altogether, our results demonstrate strain-specific effects of commensal *E. coli* in the exacerbation of acute inflammation and development of the pro-inflammatory immune responses that drive intestinal tissue damage.

## Methods

### Bacterial strains

Four *E. coli* strains were isolated from the feces of specific pathogen-free, healthy conventional microbiota mice and are described in Table 1. Strain SWW33 was a kind gift from Dr. Michael Wannemuehler at Iowa State University. Strains CEC-1, CEC-7, and CEC-8 were isolated from mice housed at the University of Nebraska-Lincoln. For *E. coli* strain isolation, fecal pellets were resuspended in sterile 1X PBS (HyClone, Logan, Utah, USA), plated on Eosin Methylene Blue (EMB) agar plates (Difco, Sparks, MD, USA) and incubated for 24 h at 37°C. Individual colonies were biochemically analyzed for *E. coli* identification as previously descried (17). All strains were stored in Brucella broth (Difco) with 20% (v/v) glycerol (Thermo FisherScientific, Middletown, PA, USA) at −80°C. To further characterize these four strains and determine whether they were derived from unique populations, the strains were subjected to O and H serotyping using either antisera generated against each *E. coli* O-serogroup or PCR-RFLP analysis for H typing, respectively, as previously described (18, 19). Strains were also analyzed by multilocus sequence typing (MLST) utilizing the sequences of eight housekeeping genes as described in the Pasteur *E. coli* MSLT scheme (20). Allelic sequences at the eight loci were then used to define the sequence type (ST) of each isolate (21). *E. coli* phylogrouping for each strain was determined using the triplex PCR described by Clermont et al. (16). For mouse experiments, *E. coli* strains were plated on EMB agar plates, grown overnight at 37°C and used to inoculate autoclaved Luria Bertani (LB) broth composed of 5 g bacto tryptone (Difco), 2.5 g yeast extract (Difco) and 5 g sodium chloride (Thermo FisherScientific) dissolved in 500 mL deionized water. Cultures were grown overnight at 37°C without shaking. The next day, 1 mL of each overnight culture was transferred to a fresh 10 mL culture of LB broth and incubated at 37°C for 4–6 h (to late log phase) with shaking at 200 rpm. Bacterial cultures were then centrifuged and resuspended at an approximate concentration of 1 × 10^8^ colony forming units (CFU) in 200 μL of LB broth for oral gavage.

**Table 1:** MLST, conventional serotyping and phylogrouping of *E. coli* strains used in this study.

***E. coli* strain**	**Sequence type (ST)**	**O serotype**	H type^*^	Phylogroup^**^	**Source**
CEC-1	129	2	6	B2	This study
CEC-7	150	120	1	B2	This study
CEC-8	468	1	+	B1	This study
SWW33	375	110	+	B2	Michael Wannemuehler

*CEC, commensal E. coli*.

* *+ for H type indicates fliC gene present but RFLP pattern does not match known patterns*.

** *E. coli phylogroup was determined for each strain using triplex PCR described by Clermont et al. (16). based on the presence/absence of three genes (chuA, YjaA, TspE4.C2)*.

### Mice

Eight-to ten-week-old male and female C3H/HeN mice harboring the altered Schaedler flora (ASF) from birth were bred and maintained under gnotobiotic conditions at the University of Nebraska-Lincoln Gnotobiotic Mouse Facility. Members of the ASF community include: ASF 356, *Clostridium* sp.; ASF 360, *Lactobacillus intestinalis*; ASF361, *Lactobacillus murinus*; ASF 457, *Mucispirillum schaedleri*; ASF 492, *Eubacterium plexicaudatum*; ASF 500, *Pseudoflavonifractor* sp.; ASF 502, *Clostridium* sp.; and ASF 519, *Parabacteroides goldsteinii* (22). All mice were raised in flexible film isolators and, immediately prior to inoculation with *E. coli* strains, were transferred to a positive-pressure individual ventilated caging (IVC) system (Allentown Inc., Allentown, NJ, USA) and maintained as previously described (23).

ASF-bearing mice were inoculated via oral gavage with 10^8^ CFU of one of four commensal *E. coli* strains in 200 μL of LB broth. Ten days later, feces were collected from all mice, homogenized in sterile PBS, plated on EMB agar and incubated overnight at 37°C to check for successful *E. coli* colonization and to confirm the absence of *E. coli* in control mice. Intestinal inflammation was induced in mice by providing 2.5% (w/v) dextran sulfate sodium salt (DSS; MW = 36,000–50,000, MP Biomedicals, Solon, OH, USA) in drinking water for 5 consecutive days as previously described (23). This treatment was followed by a recovery period where mice received regular drinking water for 4 days prior to necropsy. All procedures involving animals were approved by the Institutional Animal Care and Use Committee at the University of Nebraska-Lincoln (Protocols 817 and 1215).

### Bacterial counts

*E. coli* colonization levels were assessed as previously described (24). Feces were collected from mice on day 0 (prior to starting DSS treatment), on day 5 (after completing DSS treatment), and on day 9 (one day prior to necropsy). Fecal samples were homogenized, diluted with sterile 1X in a series of 10-fold serial dilutions and plated on EMB agar in 10 μL volumes in triplicate. Plates were incubated at 37°C for 24 h, and the colony-forming units (CFU) present in each 10 μL drop were counted, averaged among the triplicates and the data expressed as log_10_ CFU/g of feces.

### Macroscopic and histopathological scores

Macroscopic inflammatory changes in the cecum were scored at necropsy as previously described (23, 25, 26). Macroscopic scores were assigned on a scale of 0 or 1 for the absence or presence, respectively, of each of the following criteria: cecal atrophy, enlarged cecal tonsil, emptying, presence of mucoid or watery contents, and presence of luminal blood in cecum. Individual scores were combined to generate a gross cecal score (maximum score of 5). For histopathological analysis, cecal tissues were collected at necropsy and fixed in 10% neutral buffered formalin (Thermo Fisher Scientific). Fixed tissues were then embedded in paraffin, sectioned, and stained with hematoxylin and eosin (H&E) at the Iowa State University Comparative Pathology Core (Ames, IA). Tissues were scored in a blinded fashion by a board-certified veterinary pathologist (JMH) based upon previously published parameters (23, 25, 26), which included mucosal height, gland hyperplasia, stromal collapse, edema, inflammation, and ulceration. Each parameter was scored on a scale of 0–5 according to the degree of severity and used to generate a total histopathological score ranging from 0 (no score) to 30 (maximum histopathological lesion score).

### Cecal explant culture and cytokine measurement

All reagents were obtained from Corning except for the 2-Mercaptoethanol solution, which was purchased from Thermo Fisher Scientific. For explant cultures, cecal tissues were prepared as previously described (23, 24). Culture supernatants were collected and stored at −20°C until analysis. Chemokine and cytokine levels in explant supernatants were measured using customized Mouse Cytokine/Chemokine Milliplex Magnetic Bead Kits (Millipore, Billerica, MA, USA) and a MAGPIX instrument (Luminex Corporation, Austin, TX, USA) according to the manufacturer's instructions. Please see Supplementary Material for methods describing cell culture, infection assays, isolation of intestinal epithelial and lamina propria cells, and flow cytometric analyses.

### *In vivo* IL-6 neutralization

Mice received an intraperitoneal injection of 300 μg of either anti-IL-6 mAb (clone MP5-20F3) or rat IgG1 isotype control (clone TNP6A7) 1 h prior to DSS treatment (day 0). Injections were repeated on day 2, 4, 6, and 8. Injections were delivered in a volume of 200 μL dilution buffer. All reagents were purchased from Bio X Cell (West Lebanon, NH, USA).

### Statistical and computational analyses

A portion of the analyses were performed using GraphPad Prism 6 (version 6.01, 2012, GraphPad Software, CA, USA). Data analyzed via those methods are presented as mean ± SEM. Non-normally distributed data were analyzed using a non-parametric Kruskal–Wallis test followed by unpaired Mann–Whitney *post-hoc* test unless stated otherwise in figure legends. For all experiments, *p* < 0.05 was considered statistically significant (^*^p < 0.05, ^**^p < 0.01, ^***^p < 0.001, and ^****^p < 0.0001).

To assess which cytokine was the best predictor of severe disease, a binary classification (0 or 1), was inserted into the dataset. Specifically, *E. coli* ST129-DSS and ST325-DSS treatments were classified as 1 and the remaining treatments as 0. The choice of classification was based on the results of cecal gross and histopathological scores, which together demonstrated that the *E. coli* ST129-DSS and ST325-DSS treatments induced severe disease. This approach allowed for the application of supervised machine learning methods and logistic regression models to identify the best predictors of disease. Initial screening for potential predictors was done using pairwise Spearman correlation coefficients (*p* < 0.001) to identify the cytokines that were significantly and positively associated with disease (i.e., cecal gross and histopathological scores). The correlation matrix was produced using the cor.mtest function() and corrplot () packages. Then, Random Forest [randomForest() package] and binomial model-based [glmulti() package] searching algorithms were used to identify the best predictors of disease using the binary outcome. The Random Forest selection of the most important biomarker was done based on the highest value for the decreased mean in the Gini coefficient. The binomial algorithm randomly iterated through combinations of models, including all or subsets of cytokines until it converged to a final, best-predictive model based on the Bayesian Information Criteria (BIC). BIC was used for model selection to identify the most parsimonious solution across all sets of estimated models.

After selecting the best model (which only included IL-6 as the predictor variable) based on both the Random Forest and logistic regression procedures, a generalized mixed model approach using a binomial distribution (link = logit) was used to estimate the probability of disease for the two treatments [glm() function]. The differences in probabilities between the treatments classified as either 0 or 1 were tested using the Welch two-tailed *T*-test (*p* < 0.05) [t.test() function]. The overall accuracy of this model was calculated using a cross-validation approach combined with a ROC analysis [ROCR() package]. These analyses were performed with R software, version 3.4.3 (R Core Team 2016, R Foundation for Statistical Computing, Vienna, Austria).

## Results

### Exacerbation of intestinal inflammation by commensal *E. coli* isolates was strain-specific

**Figure 1:**
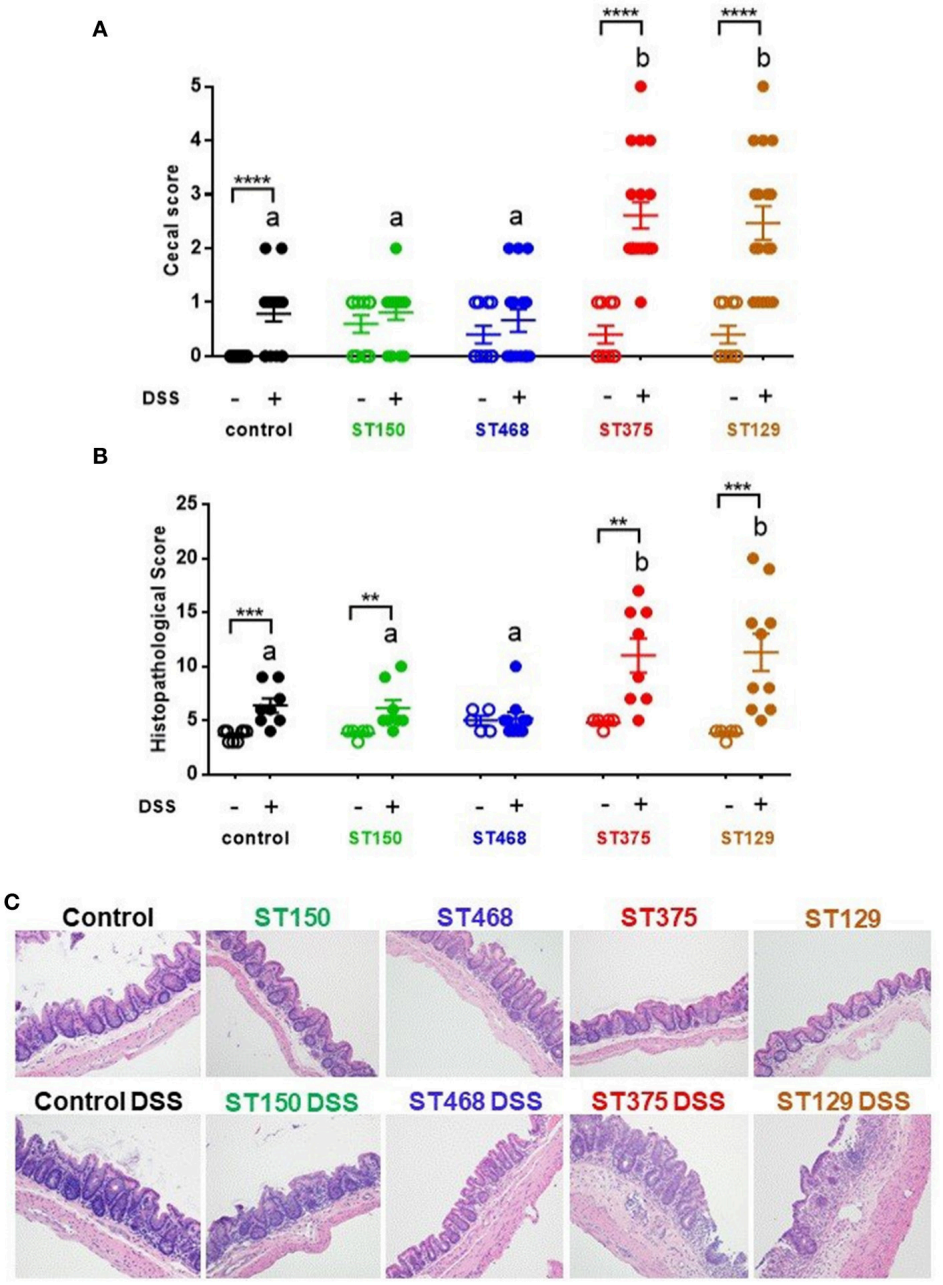
Exacerbation of intestinal inflammation by commensal *E. coli* isolates was strain-specific. **(A)** Gross cecal lesion scores for control C3H/HeN ASF mice or mice colonized with either *E. coli* ST150, ST468, ST375, or ST129 for 3 weeks (*n* ≥ 10, each group). DSS-treated mice received 2.5% DSS in their drinking water for 5 days and then regular water for 4 days. All mice were euthanized at day 10. **(B)** Histopathological cecal lesion scores for mice described in **(A)** (*n* = 5–10 animals per group). Scoring parameters included mucosal height, epithelial cell ulceration, severity of inflammatory cell infiltrate, edema, stromal collapse, and goblet cell depletion. **(C)** Representative cecum photomicrographs (H&E stained) were taken at x20 objective magnification. **(A,B)** Data were analyzed using a non-parametric Kruskal–Wallis test followed by an unpaired Mann–Whitney *post-hoc* test. Values represent the mean ± the SEM. Treatments with different letters are significantly different from one another at *P* < 0.05. **P < 0.01, ***P < 0.001, ****P < 0.0001.

To evaluate the disease-causing potential of commensal *E. coli* strains, gnotobiotic C3H/HeN mice harboring the altered Schaedler flora (ASF) were colonized with one of four gut commensal *E. coli* strains for 3 weeks prior to inducing acute intestinal injury with 2.5% DSS. We selected ASF mice for these studies because they contain a microbiome devoid of any *Proteobacteria* and can be stably colonized with *E. coli* without the need for antibiotic pretreatment. Control ASF mice (no *E. coli*) and ASF mice colonized with either *E. coli* ST150 or ST468 exhibited no to mild visible cecal inflammation when exposed to 2.5% DSS. In contrast, colonization with *E. coli* strains ST129 or ST375 caused severe cecal disease characterized by cecal atrophy and the presence of luminal blood (Figure 1A). Microscopic analysis of cecal tissues confirmed the differential disease phenotypes observed macroscopically (Figure 1B). In particular, lesions from mice colonized with either *E. coli* ST129 or ST375 and treated with DSS were characterized by extensive epithelial ulceration, submucosal and mucosal edema, neutrophilic, and monocytic cell infiltration, gland hyperplasia and stromal collapse (Figure 1C). Consistent with macroscopic observations, mice receiving either DSS only or *E. coli* strains only (but not both) developed minimal microscopic lesions (Figures 1B,C). Together, these results demonstrate that commensal *E. coli* populations have strain-specific, disease-causing potentials during intestinal inflammation.

### The abundance of commensal *E. coli* strains did not change during intestinal inflammation and was not associated with disease severity

**Figure 2:**
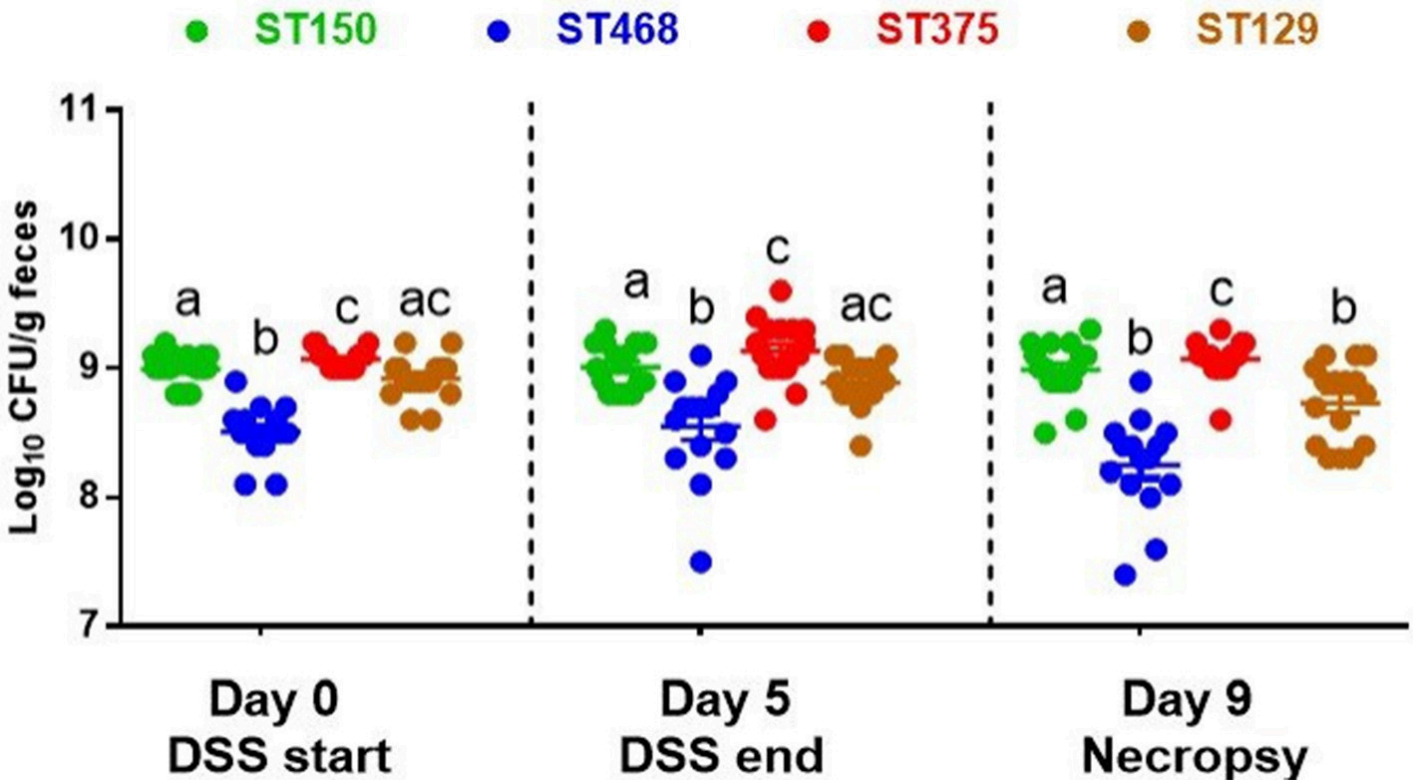
The abundance of commensal *E. coli* strains did not change during intestinal inflammation. Feces were collected form all *E. coli*-colonized mice on day 0 (before DSS treatment), day 5 (after DSS treatment), and day 9 (day before necropsy), homogenized and serially-diluted in sterile PBS. Bacterial abundance was determined by plating on EMB agar and incubating at 37°C for 24 h. Each dot represents an individual animal (*n* = 15–17). Values were log_10_ transformed and data are shown as mean ± SEM. A non-parametric Kruskal–Wallis test followed by unpaired Mann–Whitney *post-hoc* test were used to analyze data from multiple treatments at each time point separately (day 0, day 5, and day 9). The same analysis was performed to analyze data from multiple time points for the same treatment. Treatments with different letters are significantly different from one another at *P* < 0.05.

Several studies have reported that increases in the abundance of *Enterobacteriaceae*, including *E. coli*, often occur during intestinal inflammation (27–29). To determine whether the enhanced intestinal inflammation caused by *E. coli* ST129 and ST375 was accompanied by an increase in their abundance, all *E. coli* strains were quantified in the feces of mice immediately prior to initiating DSS treatment (day 0), after DSS treatment (day 5), and one day prior to necropsy (day 9). Across all time points evaluated, the average abundance of each *E. coli* strain ranged from 10^8^ to 10^9^ (Figure 2). Further, levels of *E. coli* ST129 (disease-causing) and ST150 (no disease) were not significantly different from one another. An evaluation of each strain's abundance both before and after DSS exposure also revealed no significant changes except for a slight decrease in the abundance of ST129 (disease-causing) at necropsy (Figure 2). Altogether, these findings demonstrate that commensal *E. coli*-mediated intestinal inflammation was not associated with notable increased abundances of these strains either prior to or after DSS exposure.

### Strain-specific mucosal pro-inflammatory responses were observed during intestinal injury of mice colonized with commensal *E. coli* isolates

**Figure 3:**
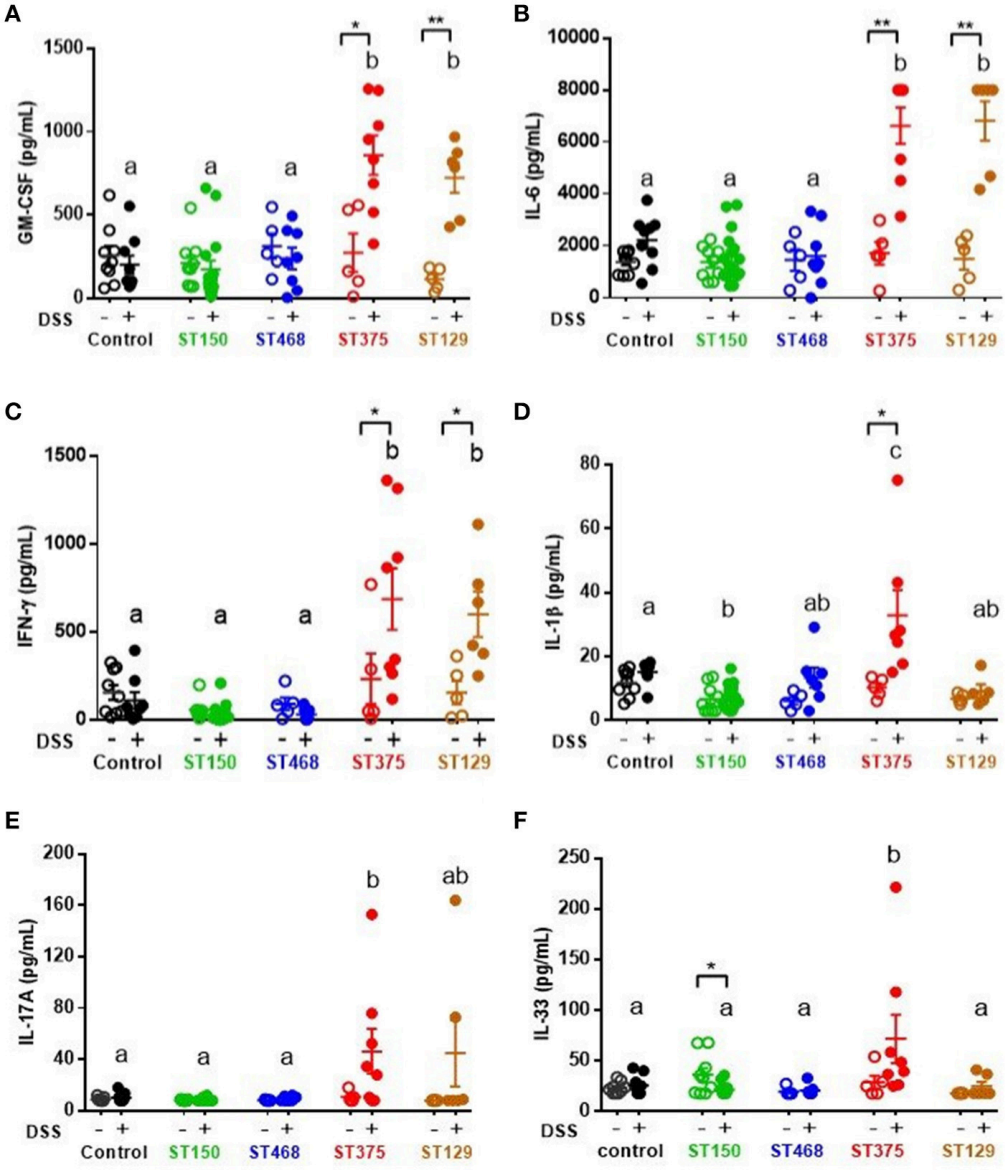
Strain-specific mucosal pro-inflammatory responses were observed during intestinal injury of mice colonized with commensal *E. coli* isolates. Cecal explant tissues from control ASF mice or ASF mice colonized with either commensal *E. coli* ST150, ST468, ST375, or ST129 were cultured overnight. Supernatants from these cultures were assessed by a magnetic multiplex assay (*n* = 5–16 animals per group) for levels of **(A)** GM-CSF, **(B)** IL-6, **(C)** IFN-γ, **(D)** IL-1β, **(E)** IL-17A, and **(F)** IL-33. All data were analyzed by a non-parametric Kruskal–Wallis test followed by unpaired Mann–Whitney *post-hoc* test. Values represent the mean ± SEM. Treatments with different letters are significantly different from one another at *P* < 0.05. *P < 0.05, **P < 0.01.

Because disease development was found to be independent of *E. coli* abundance, we hypothesized that the exacerbated intestinal inflammation caused by *E. coli* ST129 and ST375 was instead due to the induction of a more robust pro-inflammatory host immune response during intestinal injury compared to ST150 and ST468. To address this possibility, levels of cytokines were evaluated in cecal explant cultures. Following DSS exposure, cecal explants from mice colonized with either *E. coli* ST129 or ST375 (disease-causing) produced significantly higher levels of the pro-inflammatory cytokines GM-CSF, IL-6, and IFN-γ compared to control mice or mice colonized with ST150 or ST468 (no disease) (Figures 3A–C). Cecal explants from mice colonized with *E. coli* ST375 and treated with DSS also secreted elevated levels of the pro-inflammatory cytokines IL-1β, IL-17A, and IL-33 relative to other treatments (Figures 3D–F).

**Figure 4:**
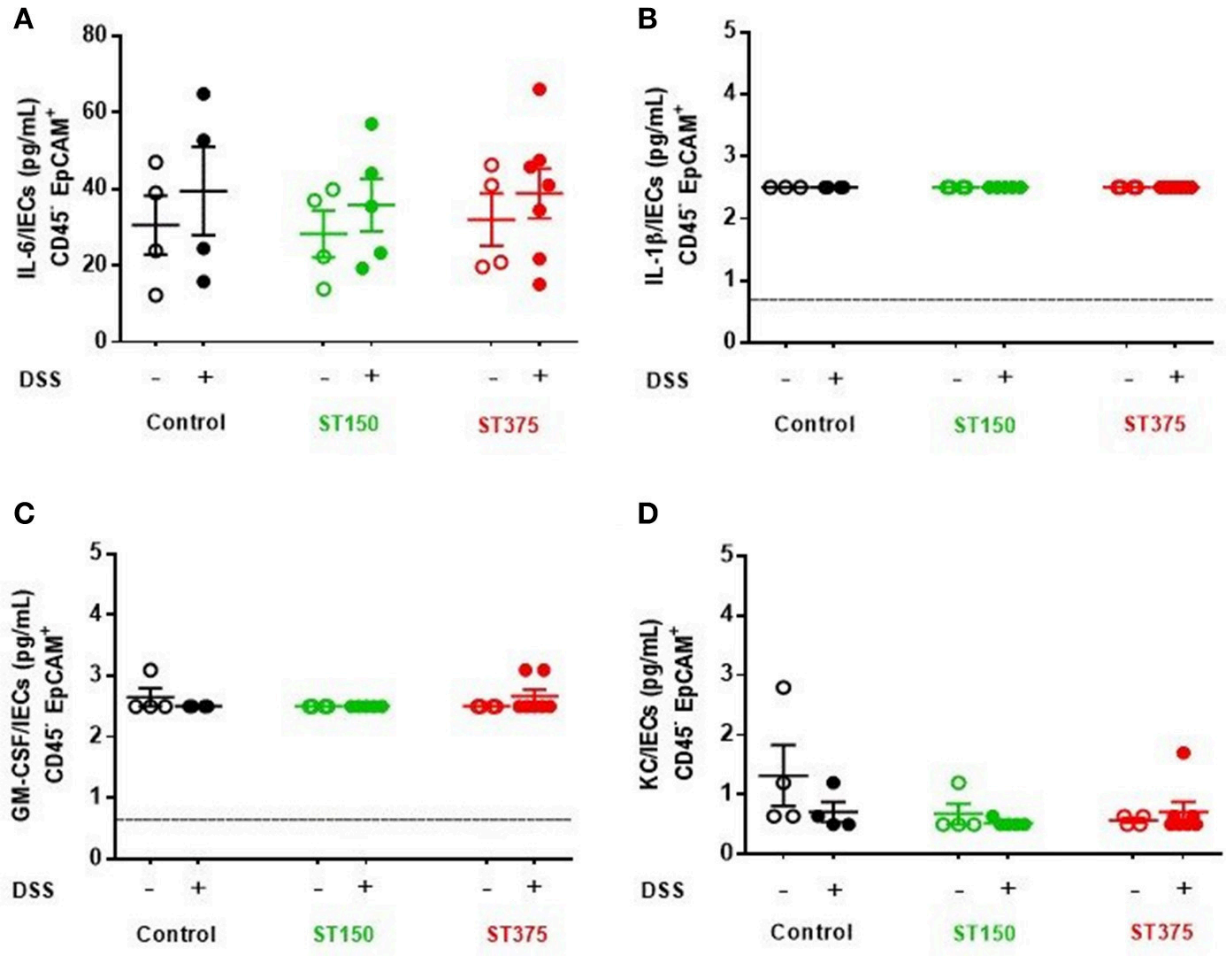
Commensal *E. coli* strains induced production of similar pro-inflammatory cytokine profiles from intestinal epithelial cells. Purified intestinal epithelial cells (CD45^−^ EpCAM^+^) were sorted from ASF mice, either control or colonized with *E. coli* ST150 or ST375. Cells were cultured for 3 hr and the supernatants assessed for levels of **(A)** IL-6, **(B)** IL-1β, **(C)** GM-CSF, and **(D)** KC using a magnetic multiplex assay (each dot is a pool of 2–3 mice). Values represent mean ± SEM. **(B,C)** Vertical dashed lines represent assay detection level (0.64 pg/mL). Data were analyzed using a non-parametric Kruskal–Wallis test followed by unpaired Mann–Whitney *post-hoc* test.

**Figure 5:**
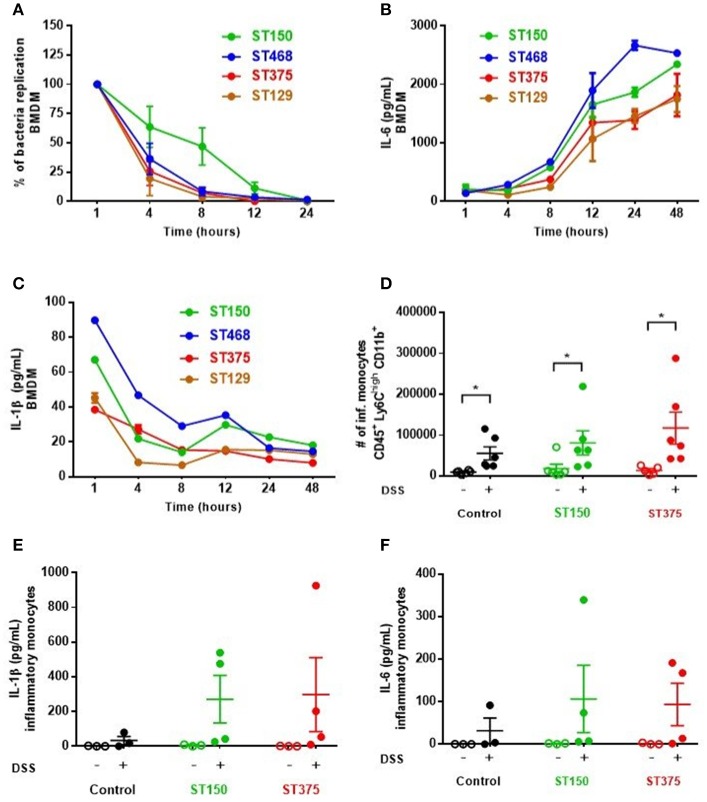
Commensal *E. coli* strains elicited similar pro-inflammatory cytokine profiles from monocytes and antigen presenting cells. **(A–C)** Bone marrow-derived macrophages (BMDM) were seeded and plated for 24 h prior to infection with *E. coli* strains at MOI 10 **(A)** Survival and replication of each strain was assessed by lysing macrophages and plating on EMB agar plates. The percentage of intracellular bacteria was graphed relative to that obtained 1 h-post gentamicin treatment (defined as 100%). **(B,C)** Culture supernatants from infected BMDM were analyzed to measure IL-6 and IL-1β levels via ELISA. Values are the mean of triplicate samples ± SEM of at least two experiments. **(D)** Number of recruited inflammatory monocytes (Ly6C^high^ CD11b^+^) in isolated CD45^+^ LP cells were determined by flow cytometry. Levels of IL-1β **(E)** and IL-6 **(F)** present in culture supernatants of inflammatory monocytes were assessed by a magnetic multiplex assay (each dot is a pool of 2–3 mice). Data are shown as the mean ± SEM. For **(A–C)**, multiple unpaired *t-*tests were performed on each time point indicated. For **(D–F)**, a non-parametric Kruskal–Wallis test followed by unpaired Mann–Whitney *post-hoc* test was used to analyze the data. *P < 0.05.

*Ex vivo* analyses aimed at assessing the specific role of intestinal epithelial cells (IEC) in pro-inflammatory cytokine production revealed no differences in cytokine secretion from CD45^−^ EpCAM^+^ IEC sorted from the ceca of control and DSS-treated mice colonized with either *E. coli* ST150 or ST375 (Figure 4). Furthermore, *in vitro* infection of bone marrow-derived macrophages with *E. coli* strains ST150 and ST375 induced comparable IL-6 and IL-1β production (Figure 5). We next isolated CD45^+^ lamina propria cells from the ceca of mice colonized with either *E. coli* ST150 or ST375 ± DSS treatment. We observed both similar numbers of, and pro-inflammatory cytokine production from, Ly6C^low^ CD11b^+^ phagocytic cells, Ly6C^high^ CD11c^+^ CD11b^−^ dendritic cells, and Ly6C^high^ CD11b^+^ recruited inflammatory monocytes (Figure 5 and Supplementary Figures S1–S3). Other analyses revealed no detectible antigen-specific IFN-γ in cultures of purified CD4^+^ T cells isolated from the mesenteric lymph nodes of mice colonized with *E. coli* ± DSS treatment when stimulated with antigens from the commensal *E. coli* strains or the ASF community (Supplementary Figure S4). Together, these results demonstrate that gut commensal *E. coli* strains can induce differential mucosal cytokine production. However, such differential responses are not attributable specifically to IEC, monocytes or CD4^+^ T cells.

### Mucosal IL-6 production best predicted commensal *E. coli*-mediated intestinal inflammation

**Figure 6:**
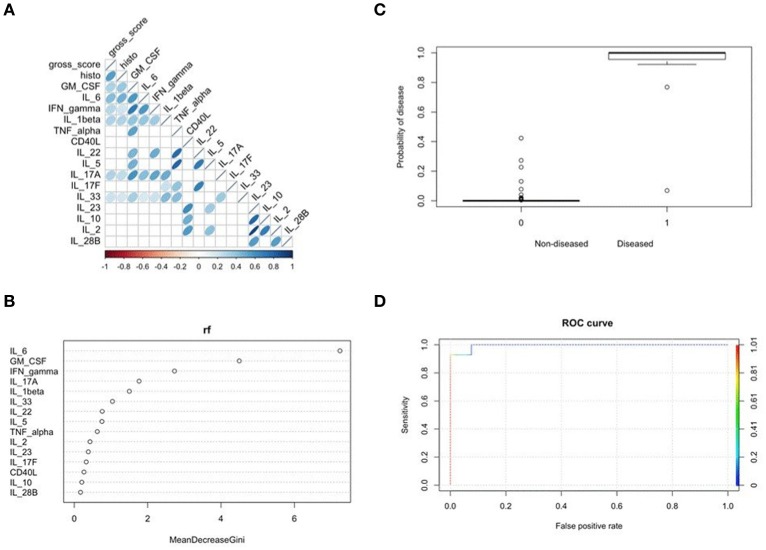
Mucosal IL-6 production best predicted *E. coli* ST129- and ST375-mediated intestinal inflammation. **(A)** Spearman correlation plot depicting all pairwise correlations between cytokines/chemokines and disease scores. The darker the shade of blue and the thinner the ellipse, the stronger the correlation coefficient. Correlation coefficients were considered to be significant at *p* < 0.001 to account for multiple comparisons. **(B)** Random forest analysis was used to assess which cytokine was the best sole predictor of disease occurrence when grouping all treatments as either 0 (no to mild disease) versus 1 (moderate to severe disease). Specifically, *E. coli* ST129-DSS and ST325-DSS treatments were classified as 1 and the remainder treatments as 0. The strongest biomarker of disease was determined based on the highest mean decrease in the Gini coefficient. **(C)** Results from a binomial model using IL-6 as the independent variable to predict the probability of disease using the binary classification described above in **(B)** (i.e., 0 or 1 for the dependent variable). Plot shows a significant increase in the probability of disease for *E. coli* ST129-DSS and ST325-DSS treatments (*p* = 0.025). **(D)** Cross-validation analysis to determine the accuracy of the binomial model for classifying between groups coded as either 0 or 1 when using IL-6 as the only predictor. ROC analysis shows an overall 97.5% accuracy of the model-based classification between the two treatments.

To determine which cytokine(s) produced by cecal explants best predicted disease outcome, we combined results from a Spearman correlation analysis with a Random Forest and binomial algorithm BIC-based selection and model estimation. Collectively, this approach allowed calculation of disease probability associated with a given biomarker. Correlation analysis identified several cytokines, including IL-6, as significantly and positively correlated with disease scores (Figure 6A). Supervised machine learning and searching algorithms also identified IL-6 as the best predictor of disease categorized as either 0 (no to mild disease) or 1 (moderate to severe disease) (Figure 6B). Importantly, this binary score was specifically chosen as a classifying variable to separate the most severely diseased *E. coli* ST129- and ST375-colonized mice treated with DSS from the other treatments. A binomial model was then used to assess the relationship of IL-6 levels on the probability of predicting disease. Elevated IL-6 levels in cecal explants not only significantly affected disease prediction (*p* = 0.025), but clearly increased the likelihood of disease development in both *E. coli* ST129- and ST375-colonized mice treated with DSS (categorized as 1) (Figure 6C). Finally, cross-validation-based model estimation and ROC analysis revealed an overall accuracy of 97.5% (specificity = 98.48% and sensitivity = 92.86%) in classifying mice between the two treatments (0 vs. 1) (Figure 6D). Together, these findings demonstrate that during intestinal injury, pro-inflammatory responses—especially IL-6 production—are associated with commensal *E. coli* isolates capable of inducing severe disease.

### IL-6 blockade attenuated disease severity in mice colonized with *E. coli* ST375 and exposed to DSS

**Figure 7:**
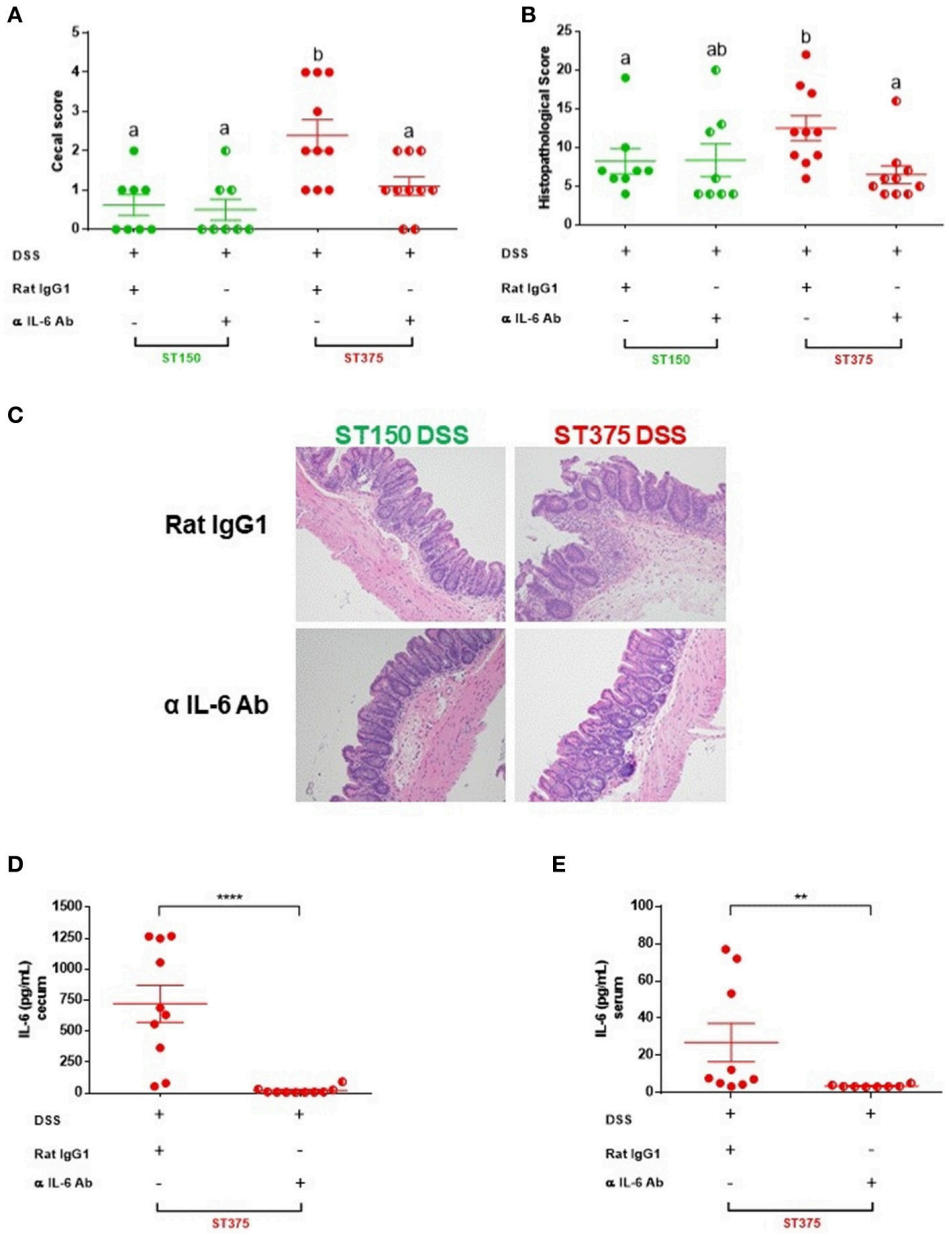
IL-6 blockade attenuated disease severity in mice colonized with *E. coli* ST375 and exposed to DSS. **(A)** Gross cecal lesion scores depicting disease severity in DSS-treated ASF mice colonized with either *E. coli* ST150 or ST375 and injected with either anti-IL-6 mAb or rat IgG isotype control. Mice received 300 μg of either anti-IL-6 or rat IgG per injection on days 0, 2, 4, 6, and 8 of DSS treatment (*n* = 8–10 per group). Histopathological scores **(B)** were assessed based upon the parameters described in Figure 1. **(C)** Representative photomicrographs of H&E stained tissues were taken at x20 objective magnification (*n* = 8–10 per group). IL-6 levels present in colonic explant cultures **(D)** and serum **(E)** were assessed by ELISA. Gross cecal score data were analyzed using a non-parametric Kruskal-Wallis test followed by an unpaired Mann–Whitney *post-hoc* test. Histopathology scores and cytokine data were analyzed with a non-parametric unpaired Mann–Whitney test. Values represent the mean ± SEM. Treatments with different letters are significantly different from one another at *P* < 0.05. **P < 0.01, ****P < 0.0001.

IL-6 has pleotropic immunological effects and can either promote intestinal inflammation (30) or provide mucosal protection (31) in different murine models of colitis. The elevated mucosal IL-6 response observed following DSS treatment of *E. coli* ST129- or ST375-colonized mice (but not in mice colonized with *E. coli* ST150 or ST468), along with the computational prediction, suggests a pivotal role for this cytokine in disease progression. To determine if IL-6 is functionally important in mediating intestinal inflammation, mice colonized with either *E. coli* ST150 or ST375 were injected intraperitoneally with either an anti-IL-6 monoclonal or rat IgG isotype control antibody prior to and during DSS exposure. Neutralization of IL-6 by the monoclonal antibody significantly reduced the severity of disease observed in *E. coli* ST375-colonized mice exposed to DSS compared to isotype control-treated mice (Figure 7). Strikingly, the macroscopic and microscopic disease scores for anti-IL-6 treated mice were comparable to mice colonized with non-disease-causing *E. coli* ST150 and exposed to DSS (Figures 7A–C), suggesting that IL-6 is a major driver of the disease response. In contrast, both anti-IL-6- and isotype control-treated mice colonized with *E. coli* ST150 and exposed to DSS failed to develop disease. An analysis of cecal explant cultures and serum from *E. coli* ST375 mice treated with anti-IL-6 and DSS revealed low to undetectable levels of IL-6 when compared to their isotype control counterparts (Figures 7D,E), suggesting successful local and systemic blockade of IL-6 production. Taken together, these results indicate that the differential production of IL-6 observed during disease caused by distinct commensal *E. coli* strains does play a critical biological role in mediating intestinal inflammation following DSS administration.

## Discussion

Immune system-microbiota interactions are highly complex but often confer mutual benefits to both the host and resident microbes (3, 4). However, during an inflammatory insult, this symbiotic alliance is altered and significant immunological and microbiological changes occur that typically culminate in immunopathology. Such alterations are often accompanied by a loss of bacterial diversity and a corresponding bloom of one or a few bacterial species, some of which may further aggravate inflammation (5, 32). During this transition to an inflammatory state, it is believed that species/strains with pathobiont potential shift from being innocuous members of the microbiota to effectors that further intensify the inflammation.

In the current study, we found that randomly chosen, freshly isolated gut commensal *E. coli* strains displayed either a neutral effect on inflammation induced by DSS or they exacerbated inflammation, ultimately leading to overt disease. The two strains with pathobiont characteristics appear to drive inflammation largely through GM-CSF, IL-6, and IFN-γ production—a cytokine signature known to actively contribute to colitis progression (30, 33, 34). The importance of the elevated cytokine profile observed in ST375- mediated intestinal inflammation was tested by an *in vivo* neutralization experiment. We focused on IL-6 neutralization to test this hypothesis based on data mining techniques, which showed that among GM-CSF, IL-6, and IFN-γ, IL-6 was the key cytokine that explained differential disease severity induced by the *E. coli* strains. Neutralization of IL-6 *in vivo* did indeed bring about significant improvements in *E. coli* ST375-mediated intestinal inflammation. Based on these findings, we propose that the elevated cecal levels of IL-6 observed in DSS-exposed mice colonized by *E. coli* ST129 and ST375 are biologically relevant, and the capacity of these *E. coli* strains to elicit high levels of IL-6 plays a critical role in promoting disease severity. Since we did not test *in vivo* neutralization of the other elevated pro-inflammatory cytokines (e.g., GM-CSF and IFN-γ), it remains entirely possible that they may also play important roles in exacerbating intestinal inflammation.

In our study, we were not able to pinpoint a specific cell type responsible for the differential mucosal IL-6 production observed among the *E. coli* strains we tested. This observation may be partly explained by previous studies reporting that IL-6 is produced by multiple cellular sources during colitis and colorectal cancer (35, 36). Lamina propria mononuclear cells isolated from colonic biopsies from ulcerative colitis and Crohn's disease patients released high levels of IL-6 when compared to control subjects (37). CD11b^+^ lamina propria macrophages have also been shown to be a source of IL-6 in DSS-treated BALB/c mice (38). Murine colonic epithelial cells also produce high levels of IL-6 following infection with *Citrobacter rodentium* (24, 31). Furthermore, Kuhn et al. have reported induction of IL-6 by intraepithelial lymphocytes in mice transgenic for dominant negative *Tgfbr2* expressed in T cells and a knockout of the *IL10rb* gene shortly after colitis development (36). Thus, it seems likely that the elevated IL-6 levels result from production by a consortium of immune and epithelial cells in mice colonized with either *E. coli* ST129 or ST375 and exposed to DSS. We also acknowledge that host genetics and the nature of the inflammatory trigger could influence immune cell involvement, cytokine profiles, and disease outcomes.

The exact characteristics of *E. coli* ST129 and ST375 that trigger these cells to respond to the inflammatory stimuli and synergistically exacerbate intestinal tissue damage during acute inflammation remain to be identified. Abundance differences can be eliminated as a factor, as the relative abundances of ST129 and ST375 strains were not different than those of ST150 and ST468. Although the cause-effect relationships are not well-defined, a common general observation among microbial imbalances during inflammatory states is decreased abundances of taxa belonging to the Firmicutes and Bacteroidetes phyla and the corresponding expansion of gram-negative bacteria (i.e., Proteobacteria phylum), especially members of the family *Enterobacteriaceae* (i.e., *E. coli*) (6, 7). Previous studies of intestinal inflammation in particular often report expansions of *E. coli* (28, 29). In contrast to these disease-associated blooms of *Enterobacteriaceae*, differential disease outcomes mediated by the ST129 and ST375 populations of *E. coli* tested in our study were not a consequence of notable differences in bacterial abundances. We observed similar *E. coli* colonization levels for each strain during all conditions, suggesting no direct association between disease severity and bacterial burden in the gut under our experimental parameters. This result may not be surprising since our gnotobiotic mice harbor a minimal bacterial community, which allows each *E. coli* strain to robustly colonize immediately upon introduction due to niche availability. The absence of abundance differences as well as the absence of abundance changes even after DSS-induced inflammation suggests that the mechanisms through which *E. coli* ST129 and ST375 exacerbate inflammation may instead involve changes in gene expression patterns or metabolic activity. Disease induction by pathobionts in the absence of abundance changes has also been reported in *Helicobacter* pathobionts. For example, *H. bilis* elicited intestinal inflammation and pro-inflammatory immune responses directed against members of the resident gut microbiota without increasing in abundance (23). Similar observations have also been reported for *H. hepaticus* (39). Thus, the capacity to induce immunopathological effects without expanding during intestinal inflammation may be a general feature of the pathobiont lifestyle, at least among the Proteobacteria.

Several lineages of frank pathogens have evolved within the species *E. coli*, mostly through acquisition of different combinations of virulence genes (10). How the pathobiont phenotype emerges in ST129 and ST375 is completely unknown. Indeed, the phenomenon has only been studied in a relatively small number of strains, and the pathobiont phenotype itself is neither well-defined nor standardized across studies. Our data in the ASF gnotobiotic mouse model shows that the phenotype of exacerbating DSS-induced inflammation can be detected in two distantly related populations of *E. coli* based on their MLST sequence types. The shared phenotypic characteristics of ST129 and ST375 include common histopathological features and cytokine signatures, suggesting the phenotypes manifest through similar mechanisms for both strains. If the mechanisms are indeed similar, then the distant evolutionary relationship of ST129 and ST375 implies that this particular pathobiont phenotype may have emerged independently in each lineage. Certainly, there is great need to understand the mechanism and the genes/alleles that bring about this pathobiont phenotype. Since we only examined a single strain of ST129 and ST375, it is not even clear if the pathobiont phenotype is shared among all or most strains of the clonal lineages marked by these STs. Moreover, it is also unknown how widespread the phenotype may be among the diverse populations of *E. coli* that have been identified.

Further supporting the idea that the pathobiont phenotype may arise on multiple occasions are findings from others who report both context-dependent and strain-specific differences in the ability of *E. coli* isolates to either protect against or cause disease. Oral administration of the putative probiotic *E. coli* Nissle 1917 is documented to be protective during DSS-induced colitis (40, 41). However, Nissle 1917 and *E. coli* K12 (a lab-adapted strain) both exacerbated intestinal inflammation and translocated to systemic organs in gnotobiotic C57BL/6j mice infected with *Toxoplasma gondii* (42). NC101, a commensal *E. coli* strain isolated from healthy mice, also exacerbated colitis when monoassociated with germ-free IL-10^−/−^ mice but not in wild-type control animals (43). Collectively, these observations demonstrate profound differential effects, both protective and pathogenic, of *E. coli* strains during intestinal inflammation.

In conclusion, our findings extend the literature regarding the strain-specific effects of *E. coli* by demonstrating the potential of unique populations of *E. coli* isolated from healthy mice to cause disease via differential induction of a pro-inflammatory cytokine response that directly contributes to intestinal tissue damage.

## Author contributions

HK and AR-T conceived the studies and designed the experiments. HK, JG-N, KH, AG, RRS, LC, LB, and RJS performed experiments. RS helped with MLST data analysis. JG-N provided multivariate cytokines data analysis. JH was the veterinary pathologist who scored tissues. AB contributed to experimental design and data interpretation. HK, AB, and AR-T wrote the manuscript with contributions from all authors.

### Conflict of interest statement

The authors declare that the research was conducted in the absence of any commercial or financial relationships that could be construed as a potential conflict of interest.
