# Supplementary material for: Commensal Escherichia coli Strains Can Promote Intestinal Inflammation via Differential Interleukin-6 Production
Source: Front Immunol. 2018 Oct 9;9:2318. doi: 10.3389/fimmu.2018.02318 (PMC6189283; doi:10.3389/fimmu.2018.02318)
Supplement: Supplementary file 1 [file Data_Sheet_1.pdf]

Supplementary Material

Commensal *Escherichia coli* strains can promote intestinal inflammation via differential interleukin-6 production

Hatem Kittana, João C. Gomes-Neto, Kari Heck, Abby Geis, Rafael R. Segura Muñoz, Liz A. Cody, Robert J. Schmaltz, Laure B. Bindels, Rohita Sinha, Jesse M. Hostetter, Andrew K. Benson and Amanda E. Ramer-Tait*

Supplementary Materials and Methods

Cell culture and infection assays

To determine *E. coli* survival in macrophages, bone marrow-derived macrophages (BMDM) from conventional C3H/HeN mice were harvested as previously described (Racoosin and Swanson, 1989; Chavez-Santoscoy et al., 2012). Twenty-four hr prior to infection, BMDM were plated at a density of 2 x 10^5 cells per well into 24-well tissue culture plates. Four hr prior to infection, BMDM were primed with *E. coli* O111:B4 lipopolysaccharide (LPS; 200 ng/mL, Sigma-Aldrich, Saint Louis, MO, USA). BMDM were infected with *E. coli* strains at a MOI of 10 for 2 hr at 37°C and 5% CO_2. After a 2 hr incubation, BMDM supernatants were collected for IL-6 and IL-1β ELISA analysis (Ready-SET-Go ELISA, eBioscience). BMDM were washed twice with 1X PBS and fresh CTCM media supplemented with gentamicin (Corning; 100 µg/mL) was added to the wells for 1 h to kill extracellular bacteria. Intracellular survival/replication was assessed at 1, 4, 8, 12, 24 and 48 hr post infection. For longer time points (4 - 48 hr), CTCM containing 100 µg/mL gentamicin was removed and replaced by media containing 20 µg gentamicin/mL. At each time point, gentamicin-containing media was removed and the survival/replication of each strain assessed by lysing the macrophages and plating on EMB agar plates as described for Caco2 cells. The percentage of intracellular bacteria at each time point was calculated relative to the one-hour time point post gentamicin treatment (defined as 100%).

Isolation of intestinal epithelial and lamina propria cells

Intestinal epithelial cells (IECs) were isolated from cecal tissues using a protocol adapted from previous publications (Flint et al., 1991; Hodgson et al., 2015). Cecal tissues were aseptically removed, cut open longitudinally, and washed several times with cold 1X PBS to free cecal contents. Tissues were cut into small pieces (2-3 mm) and transferred to cleating buffer and incubated for 45 min at 4°C with shaking. The chelating buffer consisted of 27 mM sodium citrate, 5 mM disodium phosphate (Na_2HPO_4), 96 mM sodium chloride (NaCl), 8 mM monopotassium phosphate (KH_2PO_4), 1.5 mM potassium chloride (KCl), 0.5 mM dithiothreitol (DTT), 55 mM D-sorbitol, 44 mM sucrose, 6 mM ethylenediaminetetraacetic acid (EDTA) and 5 mM ethylene glycol tetraacetic acid (EGTA), both at pH 7.3. Na_2HPO_4, KH_2PO_4, NaCl, DTT and EDTA were purchased from Thermo FisherScientific while all other reagents were obtained from Sigma-Aldrich. IECs were detached by vigorous shaking for 45 min followed by vortexing for 30 sec. Tissue debris were removed by passing the tissues and fluid through cell strainers (100 µM followed by 70 µM; Thermo FisherScientific). The flow through containing the IECs was centrifuged at 300 *x g* for 10 min at 4 °C; cell pellets were washed twice with cold 1X PBS

supplemented with 2% FBS to remove residual EDTA. IECs were then resuspended in complete RPMI medium, counted using trypan blue and analyzed via flow cytometry.

Lamina propria (LP) cells were isolated from cecal tissues using a protocol adapted from previous publications (Franchi et al., 2012; Geem et al., 2012). Tissues were cut into small pieces and washed three times with calcium- and magnesium-free (CMF) solution consisting of 10 mM HEPES buffer (Corning), 25 mM sodium bicarbonate (Sigma-Aldrich), and 2% heat-inactivated FBS in Ca^+- and Mg^+-free Hanks' balanced salt solution (HBSS; Gibco®, Thermo FisherScientific, pH 7.2). After washing, tissues were transferred into EDTA/FBS/CMF solution containing 0.1 mM EDTA and 10% heat-inactivated FBS in CMF solution. Tissues were incubated for 20 min in 37°C with shaking at 250 rpm. This step was repeated three times with the supernatants containing epithelial cells being discarded each time and adding back fresh EDTA/FBS/CMF solution. After the third wash, tissues were resuspended in RPMI medium supplemented with 5% heat-inactivated FBS, 2% Penicillin/Streptomycin, 0.1% Gentamycin (50 mg/mL), 1% of 200 mM L-Glutamine, 0.1% of a 50 mM 2-Mercaptoethanol solution and 1% of 100 mM sodium pyruvate solution for 10 min under the same conditions as the previous step. Tissues were then incubated in the same 5% FBS-RPMI medium containing 20,000 U/mL liberase (Sigma-Aldrich) and 1 mg/mL DNase I (Roche®, Sigma-Aldrich) for 45 min under the same conditions. After incubation, 100 mM EDTA was added to tissues for 5 minutes to stop the enzymatic digestion process. Tissues were then minced, washed twice, and the solution filtered through a 100 µM cell strainer using 5% FBS-RPMI medium supplemented with 2 mM EDTA. Digested samples were pelleted with centrifugation at 250 xg for 10 min at 4°C and resuspended in 20% Percoll solution (GE Healthcare, Sigma-Aldrich) layered onto 40% and 80% Percoll. LP cells were isolated from the interface of a 40/80 Percoll gradient after centrifugation at 1,000 *x g* for 30 min at room temperature. Cells were washed twice with 5% FBS-RPMI, counted using an AOPI staining solution (Nexcelom Bioscience, MA, USA) and analyzed via flow cytometry.

Flow cytometry

All antibodies were purchased from eBioscience, Inc. (eBioscience, CA, USA) with the exception of the LIVE/DEAD (L/D) fixable near-IR dead cell stain kit, which was obtained from Thermo FisherScientific. Isolated IECs and LP cells were first incubated with L/D stain diluted in PBS (1:500) for 30 minutes at 4°C in the dark. Next, surface staining was performed as previously described (Gomes-Neto et al., 2017). IECs were labeled with PE-eFluor610 anti-mouse CD45 (clone 104; 1:600 dilution) and APC anti-mouse CD326 (clone G8.8; 1:450 dilution). LP cells were labeled with PE-eFluor610 anti-mouse CD45 (clone 104; 1:600 dilution), PerCp-Cyanine 5.5 anti-mouse CD11b (clone M1/70; 1:300 dilution) and PE-Cyanine 7 anti-mouse Ly6C (clone HK1.4; 1:1000 dilution). Labeled cells were sorted using a FACSAria II instrument (BD Bioscience). Sorted $CD45^-$ $EpCAM^+$ IECs or various LP cell populations (including $CD45^+$ $CD11b^+$ $Ly6C^{high}$ inflammatory monocytes, $CD45^+$ $CD11b^+$ $Ly6C^{low}$ phagocytic cells, or $CD45^+$ $CD11b^-$ $Ly6C^{low}$ dendritic cells) were pooled from 2-3 mice per treatment. A total of 2×10^5 cells were plated in duplicate in 96-well plates for 3 hr at 37°C and 5% CO_2. Supernatants were collected for chemokine and cytokine analysis using customized Milliplex Magnetic Bead Kits as described above.

$CD4^+$ T cell stimulation

$CD4^+$ T cells were isolated and stimulated with splenic feeder cells pulsed with whole sonicated bacterial antigens from either *E. coli* or ASF bacteria as previously described (Ramer-

Tait et al., 2011; Gomes-Neto et al., 2017). Cell supernatants were collected and assessed for antigen-specific IFN-γ production via ELISA (Ready-SET-Go ELISA, eBioscience) as per manufacturer's instructions.

References

Chavez-Santoscoy, A.V., Roychoudhury, R., Pohl, N.L., Wannemuehler, M.J., Narasimhan, B., and Ramer-Tait, A.E. (2012). Tailoring the immune response by targeting C-type lectin receptors on alveolar macrophages using "pathogen-like" amphiphilic polyanhydride nanoparticles. *Biomaterials.* 33, 4762-4772. doi: 10.1016/j.biomaterials.

Flint, N., Cove, F.L., and Evans, G.S. (1991). A low-temperature method for the isolation of small-intestinal epithelium along the crypt-villus axis. *Biochem. J.* 280, 331-334. doi: 10.1042/bj2800331.

Franchi, L., Kamada, N., Nakamura, Y., Burberry, A., Kuffa, P., Suzuki, S., et al. (2012). NLRC4-driven production of IL-1 [beta] discriminates between pathogenic and commensal bacteria and promotes host intestinal defense. *Nat Immunol.* 13, 449-456. doi: 10.1038/ni.2263.

Geem, D., Medina-Contreras, O., Kim, W., Huang, C.S., and Denning, T.L. (2012). Isolation and characterization of dendritic cells and macrophages from the mouse intestine. *J. Vis. Exp: JoVE* (63): e4040. doi: 10.3791/4040.

Gomes-Neto, J.C., Kittana, H., Mantz, S., Segura Munoz, R.R., Schmaltz, R.J., Bindels, L.B., et al. (2017). A gut pathobiont synergizes with the microbiota to instigate inflammatory disease marked by immunoreactivity against other symbionts but not itself. *Sci. Rep.* 7, 17707. doi: 10.1038/s41598-017-18014-5.

Hodgson, A., Wier, E.M., Fu, K., Sun, X., Yu, H., Zheng, W., et al. (2015). Metalloprotease NleC suppresses host NF-κB/inflammatory responses by cleaving p65 and interfering with the p65/RPS3 interaction. *PLoS Pathog.* 11, e1004705. doi: 10.1371/journal.ppat.1004705.

Racoosin, E., and Swanson, J. (1989). Macrophage colony-stimulating factor (rM-CSF) stimulates pinocytosis in bone marrow-derived macrophages. *J. Exp. Med.* 170, 1635-1648. doi: 10.1084/jem.170.5.1635.

Ramer-Tait, A.E., Petersen, C.A., and Jones, D.E. (2011). IL-2 limits IL-12 enhanced lymphocyte proliferation during *Leishmania amazonensis* infection. *Cell. Immunol.* 270, 32-39. doi: 10.1016/j.cellimm.2011.03.016.

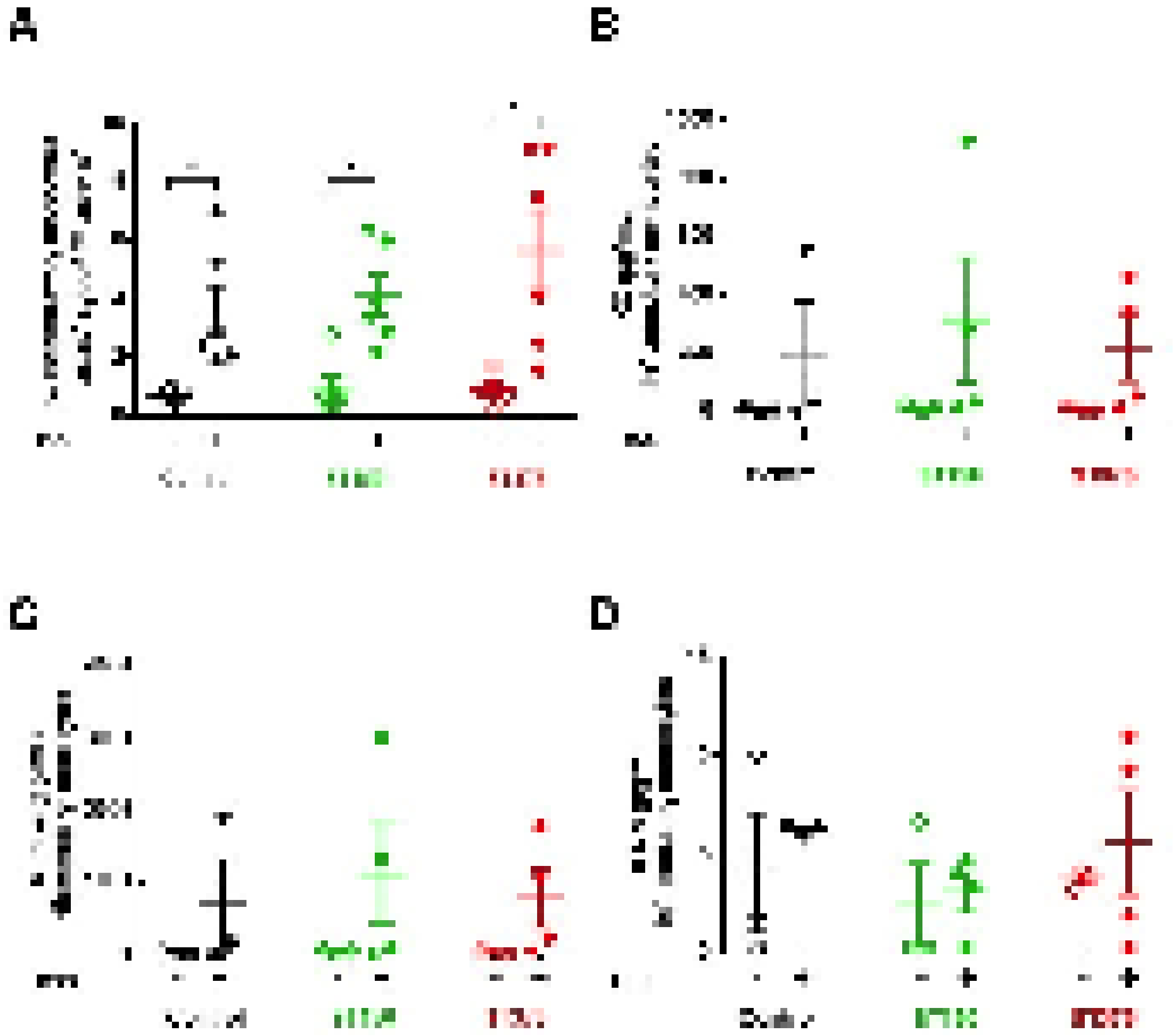

Supplementary Figure 1. Equivalent levels of pro-inflammatory cytokines were produced by recruited LP inflammatory monocytes. (A-D) Total LP cells were isolated from ASF mice, control or colonized with *E. coli* ST150 or ST375. **(A)** Percentage of recruited inflammatory monocytes (Ly6C^{high} CD11b^{+}) in isolated CD45^{+} LP cells were determined by flow cytometry. Levels of the pro-inflammatory cytokines **(B)** KC, **(C)** MIP2-α and **(D)** IFN-γ present in culture supernatants of inflammatory monocytes were assessed by Luminex assays (each dot is a pool of 2-3 mice). Data were analyzed using a non-parametric Kruskal-Wallis test followed by unpaired Mann-Whitney post-hoc test. Values represent mean ± SEM. *$P < 0.05$.

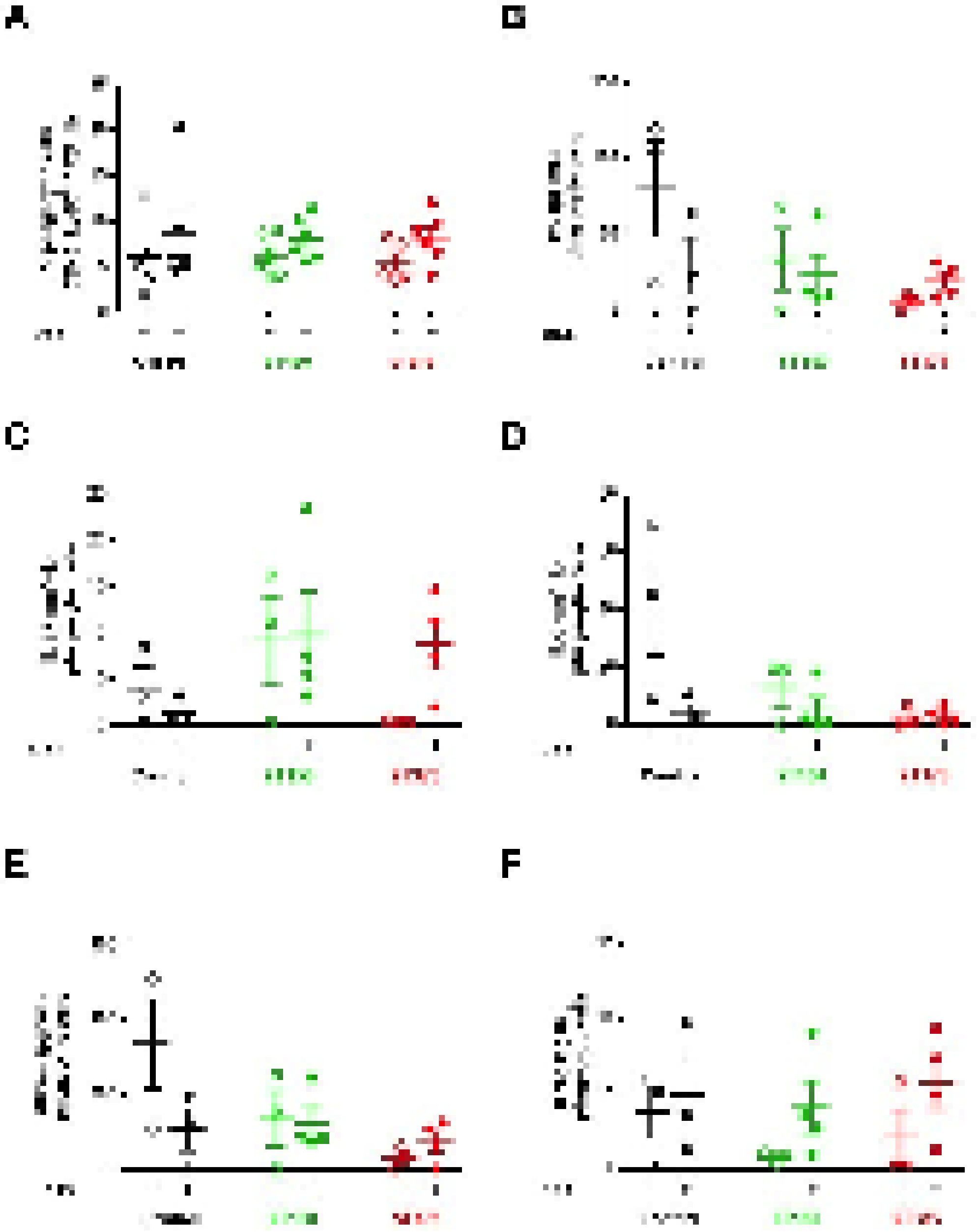

Supplementary Figure 2. Equivalent levels of pro-inflammatory cytokines were produced by phagocytic cells. (A-F) Total LP cells were isolated from ASF mice, control or colonized with *E. coli* ST150 or ST375. **(A)** Percentage of phagocytic cells (Ly6C^{low} CD11b^{+}) in isolated CD45^{+} LP cells were determined by flow cytometry. Levels of the pro-inflammatory cytokines **(B)** KC, **(C)** IL-1β, **(D)** IL-6, **(E)** MIP2-α and **(F)** IFN-γ present in culture supernatants of phagocytic cells were assessed by Luminex assays (each dot is a pool of 2-3 mice). Data were analyzed using a non-parametric Kruskal-Wallis test followed by unpaired Mann-Whitney post-hoc test. Values represent mean ± SEM.

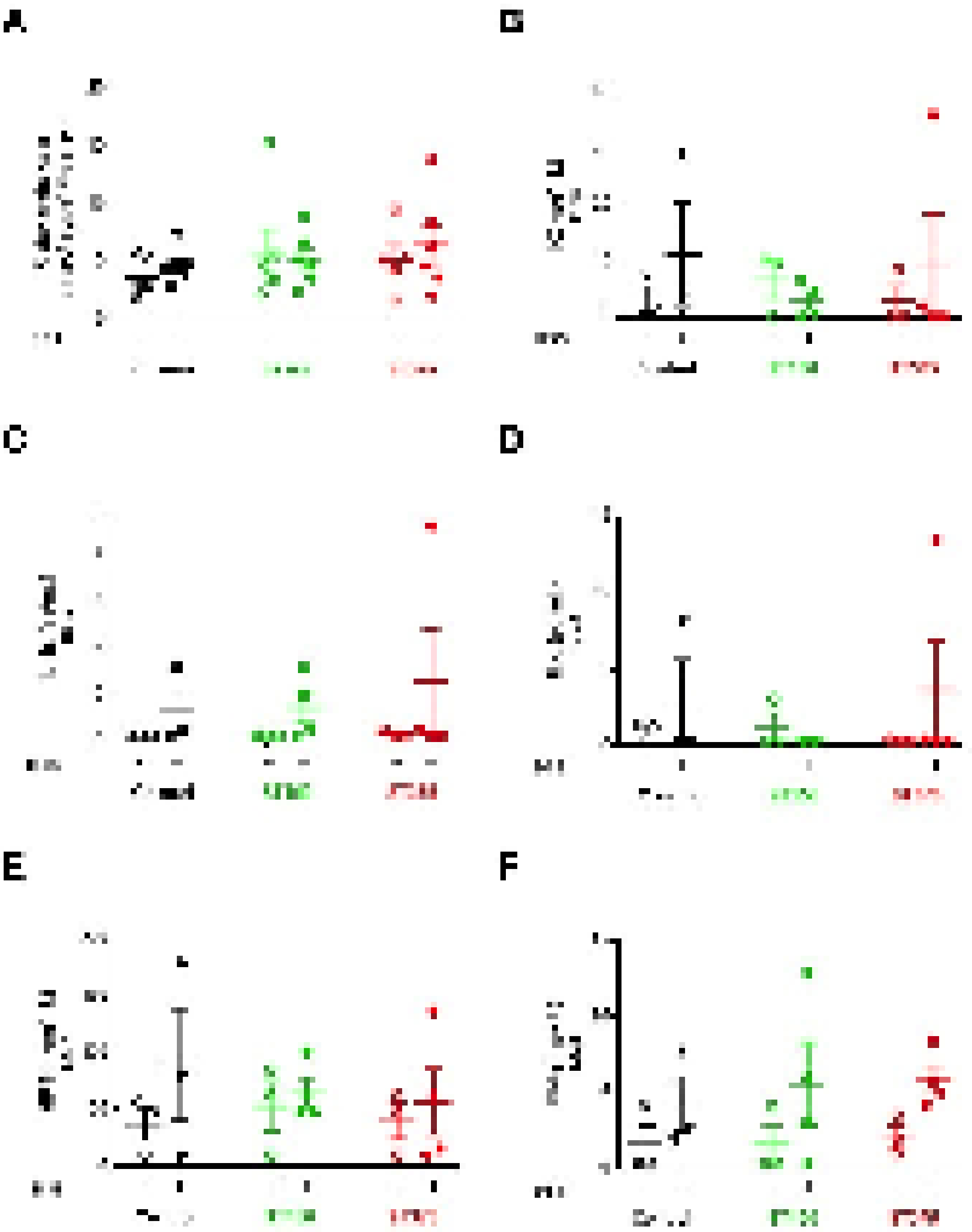

Supplementary Figure S3. Equivalent levels of pro-inflammatory cytokines were produced by dendritic cells (DCs). (A-F) Total LP cells were isolated from ASF mice, control or colonized with *E. coli* ST150 or ST375. **(A)** Percentage of DCs (Ly6C^{high} CD11c^{+} CD11b^{-}) in isolated CD45^{+} LP cells were determined by flow cytometry. Levels of the pro-inflammatory cytokines **(B)** KC, **(C)** IL-1β, **(D)** IL-6, **(E)** MIP2-α and **(F)** IFN-γ present in culture supernatants of DCs were assessed by Luminex assays (each dot is a pool of 2-3 mice). Data were analyzed using a non-parametric Kruskal-Wallis test followed by unpaired Mann-Whitney post-hoc test. Values represent mean ± SEM.

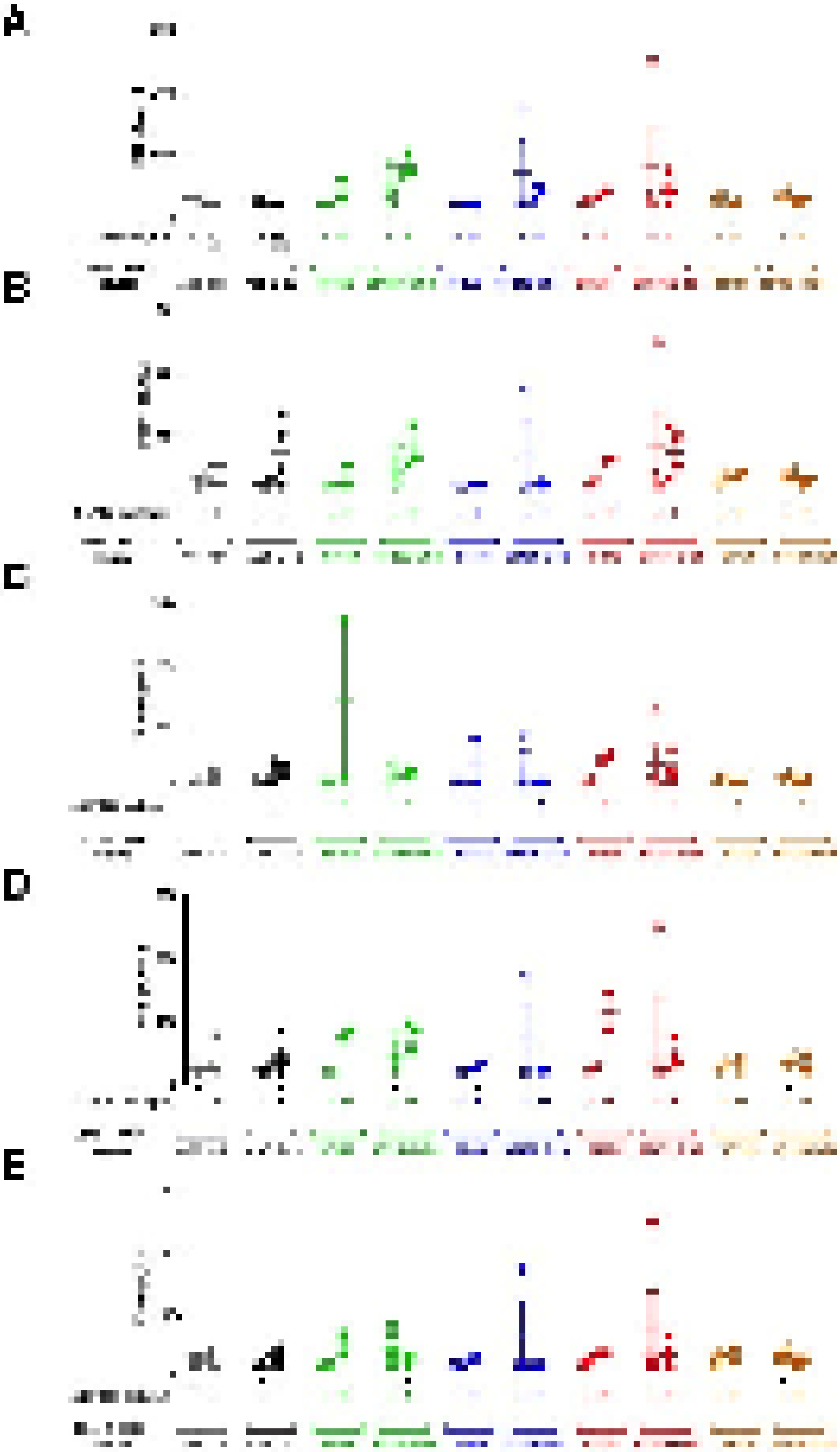
A
B
C
D
E

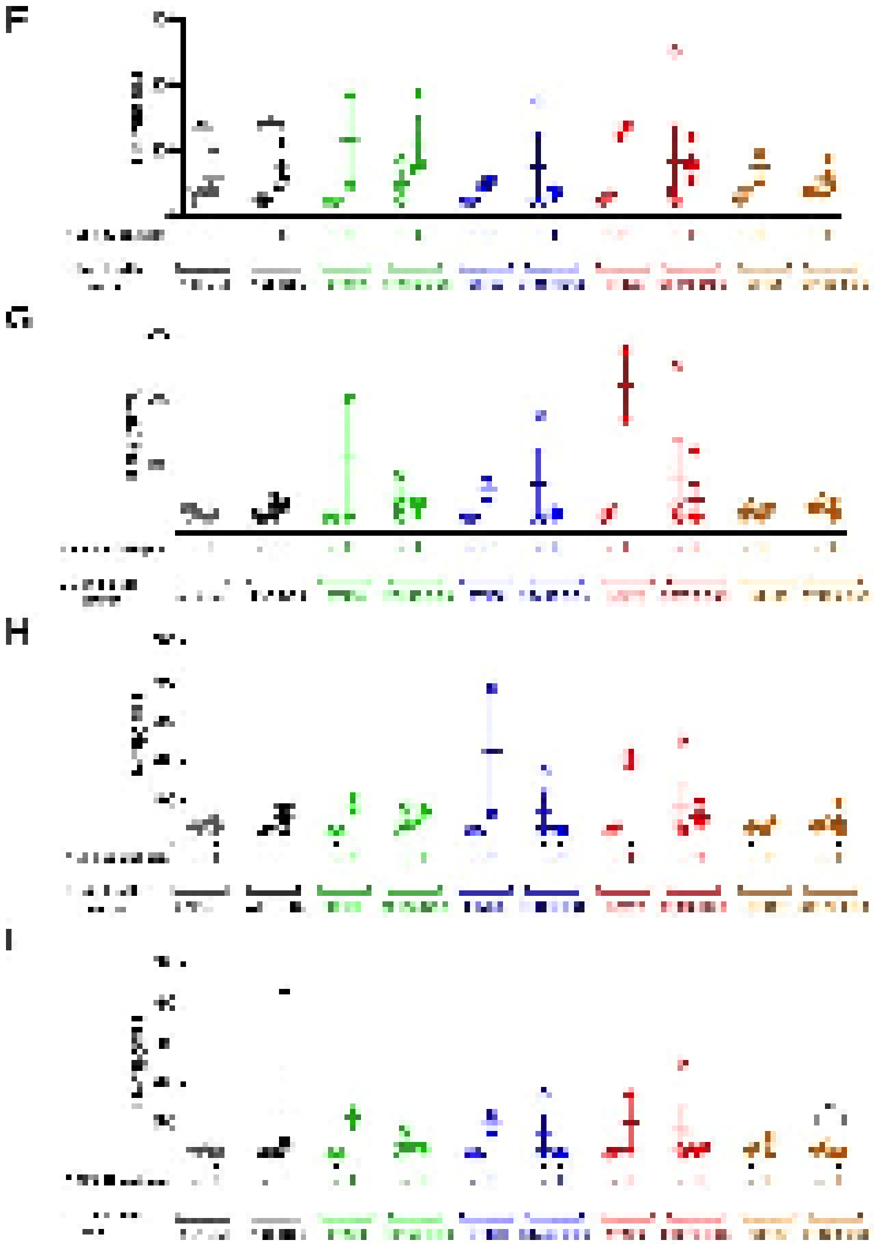

Supplementary Figure 4. No detection of antigen-specific IFN-γ CD4$^+$ T cell responses against commensal *E. coli* or ASF members. (A-I) IFN-γ production from CD4$^+$ T cells isolated from mesenteric lymph nodes of ASF mice either control or colonized with either commensal *E. coli* ST150, ST468, ST375 or ST129. CD4$^+$ T cells were either left unstimulated (NS) or stimulated with antigens from either **(A)** commensal *E. coli* ST150, ST468, ST375 or ST129, **(B)** ASF 356 (*Clostridium propionicum* class XIV), **(C)** ASF 360 (*Lactobacillus intestinalis*), **(D)** ASF 361 (*Lactobacillus murinus*), **(E)** ASF 457 (*Muscipirillum schaedleri*), **(F)** ASF 492 (*Eubacterium plexicaudatum*), **(G)** ASF 500 (*Clostridium sp*), **(H)** ASF 502 (*Ruminococcus gnavus*) or **(I)** ASF 519 (*Bacteroides distasonis*) for 72 hr at 37°C (each dot is a pool of 2-3 mice). Data were analyzed using a non-parametric Kruskal-Wallis test followed by unpaired Mann-Whitney post-hoc test. *$P < 0.05$.
